# Novel Symbiotic Association Between *Euwallacea* Ambrosia Beetle and *Fusarium* Fungus on Fig Trees in Japan

**DOI:** 10.3389/fmicb.2021.725210

**Published:** 2021-09-28

**Authors:** Zi-Ru Jiang, Hayato Masuya, Hisashi Kajimura

**Affiliations:** ^1^Laboratory of Forest Protection, Graduate School of Bioagricultural Sciences, Nagoya University, Nagoya, Japan; ^2^Department of Forest Microbiology, Forestry and Forest Products Research Institute (FFPRI), Tsukuba, Japan

**Keywords:** ambrosia *Fusarium* clade, *Euwallacea interjectus*, fig wilt disease, *Fusarium kuroshium*, *Fusarium solani* species complex, multi-gene phylogeny, mycangia

## Abstract

*Ficus carica* plantations in Japan were first reported to be infested by an ambrosia beetle species, identified as *Euwallacea interjectus*, in 1996. The purpose of this study was to determine the symbiotic fungi of female adults of *E. interjectus* emerging from *F. carica* trees infected with fig wilt disease (FWD). Dispersal adults (51 females) of *E. interjectus*, which were collected from logs of an infested fig tree in Hiroshima Prefecture, Western Japan, were separated into three respective body parts (head, thorax, and abdomen) and used for fungal isolation. Isolated fungi were identified based on the morphological characteristics and DNA sequence data. Over 13 species of associated fungi were detected, of which a specific fungus, *Fusarium kuroshium*, was dominant in female head (including oral mycangia). The plant-pathogenic fungus of FWD, *Ceratocystis ficicola*, was not observed within any body parts of *E. interjectus*. We further discussed the relationship among *E. interjectus* and its associated fungi in fig tree.

## Introduction

Ambrosia beetles are wood-inhabiting insects which cultivate fungi as a source of nutrition typically in dead, but occasionally also in healthy, woody hosts (Hulcr and Stelinski, 2017). Most of the ambrosia beetles store and transport their fungal associates to and from their natal galleries in specialized transporting organs called mycangia (singular form: mycangium) (Batra, 1963; Hulcr and Cognato, 2010; Joseph and Keyhani, 2021) or mycetangia (singular form: mycetangium) (Vega and Biedermann, 2020). Once released from the mycangia, the fungi grow in galleries to provide nutrition to the beetles and their offspring (Batra, 1963; Mueller et al., 2005; Bentz and Six, 2006). Ambrosia beetle-associated fungi play a key role in the wood by competing with the decaying fungi of the woody tissues that they infest and thereby reducing their rate of decay (Skelton et al., 2019). Many ambrosia beetles and their associated fungi are ecologically constrained to the dying or dead trees and usually remain harmless even after establishment in non-native regions (Cognato et al., 2015). Nevertheless, some invasive symbioses have been found to shift from non-pathogenic saprotrophy in native ranges to prolific tree killing in invaded ranges and cause significant damage (Hulcr and Dunn, 2011; Ranger et al., 2015; Hulcr et al., 2017; Carrillo et al., 2019; Joseph and Keyhani, 2021).

Ambrosia beetles are successful invaders (Cognato et al., 2015; Li et al., 2015; Stouthamer et al., 2017; Dzurenko et al., 2021) which exist in part due to their haplodiploid mating system, wide host range, and association with the primary ambrosia fungi, such as *Fusarium* species, which can act as both the nutritional symbionts and weak phytopathogens (Kasson et al., 2013; O’Donnell et al., 2015). There were many differences in fungal symbionts, geographic range, host preference, and potential for symbiont switching in natural populations of these beetles (Carrillo et al., 2019, 2020). *Fusarium* symbionts of ambrosia beetles belong to the genus *Euwallacea* from the monophyletic group within clade 3 of the *Fusarium solani* species complex (FSSC). It is also known as the ambrosia *Fusarium* clade (AFC; Kasson et al., 2013). Recently, some species in the FSSC, including the AFC species, were reassigned to the genus *Neocosmospora* based on gene sequences and morphology (Sandoval-Denis and Crous, 2018; Sandoval-Denis et al., 2019; Veloso et al., 2021). Previous studies have identified the phenotypic characteristics that can be used to distinguish the AFC species (Aoki et al., 2018; Lynn et al., 2021) and elucidate their host range and pathogenic potential (Eskalen et al., 2013; Kuroda et al., 2017). This provided a basis for the development, management, monitoring, and eradication strategies. However, the specific mechanism of symbiosis between the *Euwallacea* beetle and the AFC has not yet been explored. To date, only eight of the 19 AFC species in the world have been described (Table 1). It has been argued for a relatively long time that the mycangial symbionts of ambrosia beetles are strictly asexual (Mayers et al., 2017). Obligate ambrosia beetle–fungus mutualism has been represented as a one-on-one relationship (Batra, 1963). Noticeably, it has been shown in recent discoveries that the relationship between *Euwallacea* beetle and its AFC symbiosis is more likely promiscuous in native areas as opposed to strictly obligate to a specific combination of fungi which is observed in the invaded areas (Carrillo et al., 2019).

**TABLE 1:** A worldwide summary of *Euwallacea* beetle–*Fusarium* fungus symbiosis in relation to tree damage.

Beetle species	Fungal species	AFC	Host tree	Country (region)	References
*E. fornicatus*	*F. euwallaceae*	AF-2	Numerous woody hosts	Israel, United States (California, Los Angeles), South Africa	Freeman et al., 2013; O’Donnell et al., 2015; Paap et al., 2018; Smith et al., 2019
*E. fornicatus* species complex	*F. obliquiseptatum*	AF-7	Avocado (*Persea americana* Mill.)	Australia (Queensland), United States (California), Mexico	García-Avila et al., 2016; Aoki et al., 2019
*E. interjectus*	*F. floridanum*	AF-3	Box elder (*Acer negundo* L.)	United States (Florida)	Aoki et al., 2019
*E. interjectus*	*F. kuroshium*	AF-12	Fig tree (*Ficus carica* L.)	Japan	This study
*E. kuroshio*	*F. kuroshium*	AF-12	California sycamore (*Platanus racemose* Nutt.); Avocado (*Persea americana* Mill.)	United States (California), Taiwan	Gomez et al., 2018; Na et al., 2018; Carrillo et al., 2019
*E. perbrevis*	*F. ambrosium*	AF-1	Chinese tea (*Camellia sinensis* L.)	Sri Lanka, India	Gadd and Loos, 1947; Brayford, 1987; Aoki et al., 2018
*E. perbrevis*	*F. rekanum*	AF-19	*Acacia crassicarpa* A.Cunn. ex Benth.	Indonesia (Riau)	Lynn et al., 2020
*E. validus*	*F. oligoseptatum*	AF-4	Tree of heaven (*Ailanthus altissima* Mill.)	Eastern North America	Kasson et al., 2013; Aoki et al., 2018
*Euwallacea* sp.	*F. tuaranense*	AF-5	Rubber tree (*Hevea brasiliensis* Muell. Arg.)	Malaysia (North Borneo)	Aoki et al., 2019

*AFC, ambrosia Fusarium clade.*

**FIGURE 1:**
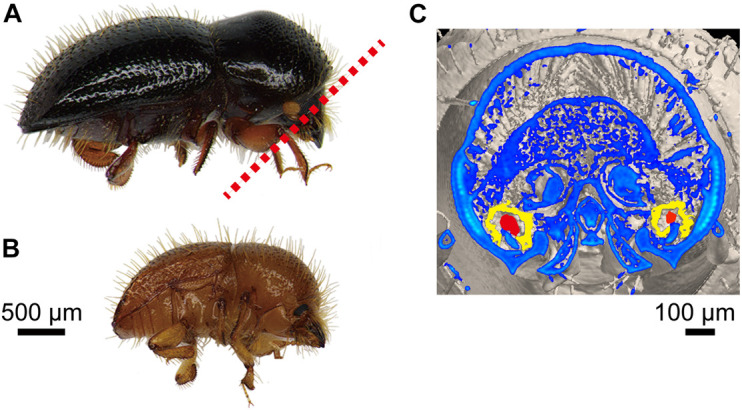
Adult *Euwallacea interjectus*. **(A)** Female and **(B)** male in lateral view. **(C)** Cross-sections of oral mycangia (colored in yellow) with compacted fungal inoculum (colored in red) in female from red dotted line in panel **(A)** by micro-computed tomography scan in Jiang et al. (2019) (unpublished image).

In Japan, the known causal agent of wilt disease on fig trees (*Ficus carica* L.) is *Ceratocystis ficicola* (Kajitani and Masuya, 2011). Fig wilt disease (FWD) has been found to be caused by the infestation of ambrosia beetle, *Euwallacea interjectus* (Blandford) (Coleoptera: Curculionidae: Scolytinae) (Kajitani, 1996, 1999; Morita et al., 2012; Kajii et al., 2013). It is a wood-boring pest of many tree species, such as poplar trees in Argentina and China and box elder (*Acer negundo* L.) in the United States (Samuelson, 1981; Aoki et al., 2019; Landi et al., 2019; Wang et al., 2020). Kajitani (1996) found that *E. interjectus* probably carried *C. ficicola* because *C. ficicola* was isolated from the fig twig into which its elytron (upper wing) was inoculated (Kajitani, 1999). In contrast, a few recent studies have discovered the presence of mycangia in the oral region (head) of female adults rather than in elytra (Li et al., 2018; Jiang et al., 2019; Figure 1). However, the role of *E. interjectus* in FWD as a suspected vector for *C. ficicola* is still unclear in Japan. It has been hypothesized that the oral mycangia of female adults might carry the fungal symbiont and/or plant pathogen to damage the host trees. Therefore, further investigation of fungal associates within the oral mycangia of *E. interjectus* would help in understanding the ecology and tree damage of the beetle species.

The purpose of this study was to investigate the beetle–fungi relationship via the identification of the recovered fungal species from the ambrosia beetle (*E. interjectus*). This was carried out by using morphology and multi-gene phylogeny, which helped in determining whether these fungal associates are pathogens of the host (fig tree). Of all the fungal associates, our aim was to isolate the fungal species present among the body parts (head, thorax, and abdomen). These were further examined for the identification of fungal species in the wild *E. interjectus* population from the diseased fig tree. This would help in clarifying the role of *E. interjectus* in spreading FWD in Japan. This study contributes to the better understanding of insect–fungus symbiosis in microbial ecology and infection by insects with fungal symbiont in applied microbiology.

## Materials and Methods

### Insect Preparation

Many pinholes made by insects were found on fig trees (variety Houraishi) infected with FWD in a fig orchard at Fukuyama, Hiroshima Prefecture, Western Japan. These insects were assumed to be *E. interjectus* based on the preliminary observation. All the insect specimens were identified by comparing the observed morphological characteristics with the previously published data (Cognato et al., 2015). This was done using an OLYMPUS SZ6045-TRPT stereomicroscope (Olympus Optical Co., Ltd., Tokyo, Japan) equipped with an OLYMPUS DP12 high-resolution microscope camera and Microsystems Digital Imaging software. Two branches, A (diameter = 11.5 cm; length = 31.5 cm) and B (diameter = 12.8 cm; length = 39.5 cm), were cut from different sections of the same infected fig tree on December 12, 2017. The infected fig tree in this study had typical FWD symptoms, showing wilting and discoloration of leaves. These branches had brown-discolored sapwood with no decay on the crosscut areas. They were stored in a cage present in the greenhouse at the Nagoya University Higashiyama Campus on December 15, 2017. During the emergence of insects from the branches, newly emerging beetle adults were sampled in the cage every 1 to 2 days. All specimens were directly kept into 1.5-ml sterile microcentrifuge tubes using disinfected forceps and transferred to the laboratory.

### Fungal Isolation and Culturing

Potato dextrose agar (PDA: 4 g potato starch, 20 g dextrose, 15 g agar, and distilled water up to 1 L) supplemented with streptomycin sulfate (100 mg/L) and synthetic low-nutrient agar (SNA: 1 g KH_2_PO_4_, 1 g KNO_3_, 0.5 g MgSO_4_⋅7H_2_O, 0.5 g KCl, 0.2 g glucose, 20 g agar, and 1 L distilled water) were autoclaved at 121°C for 15 min and were used in all the experiments. Sterile petri dishes (INA-OPTIKA Co., Ltd., Osaka, Japan) were prepared with 10 ml of PDA or SNA culture medium and kept in a sterile laminar flow chamber under UV light until the solidification of the culture medium. Fungal cultures on the PDA were used to characterize the colony and odor, whereas those on SNA were employed for the examination of microscopic characteristics.

Whole beetles were surface-washed by vortexing for 15 s in 1 ml sterile distilled water and one small drop of Tween 20 (<10 μl). A second wash was performed using sterile distilled water only. Each washed beetle was separated into body parts, viz., head, thorax, and abdomen, via two sterilized pins under an OLYMPUS SZ6045-TRPT stereomicroscope (Olympus Optical Co., Ltd., Tokyo, Japan). Afterward, the three respective body parts were inoculated individually on PDA plates. The fungi were allowed to grow (25°C, dark) for 5 to 10 days before sub-identification. The total number of colonies formed on each plate was recorded, and five colonies from each sample were selected and streaked for purification. Representative subcultures of the dominant morphotype recovered from the three respective body parts were stored on PDA slants at room temperature.

### Microscopic Observation

The specimens were initially characterized based on morphology and were grouped based on the similarities in the morphotype (such as growth speed, color, thickness, transparency, and texture). The spores were observed and photographed using a phase-contrast microscope Olympus BX41 (Olympus Optical Co., Ltd., Tokyo, Japan) and an Olympus FX380 3CCD digital camera system connected to the FLvFs software (Flovel Image Filling System version 2.30.03, Tokyo, Japan).

### Fungal Identification

At least one culture from different groups was selected for DNA extraction, including a representative from each morphological group. Fungal identification was performed based on the morphological characteristics. This data was further supported by the sequencing of the internal transcribed spacer (ITS) rDNA and ambrosia *Fusarium* sequence data of three genes, viz., translational elongation factor 1-α (TEF1) and DNA-directed RNA polymerase II largest (RPB1) and second largest subunit (RPB2).

The mycelium of each isolate was harvested from the 2-week-old plates, and their DNAs were extracted by PrepMan^®^ Ultra Sample preparation reagent (Applied Biosystems^TM^) as per the instructions of the manufacturer. Polymerase chain reaction (PCR) amplification was conducted by using these extracted DNAs and primers ITS5/ITS4 (White et al., 1990) for ITS rDNA, EF1/EF2 (O’Donnell et al., 1998) for TEF1, newly designed primers, AF-RPB1F (5′-TTCCTCACCAAGGAGCAGAT-3′)/AF-RPB1R (5′-TCGCCAATAACATGGTCAAA-3′) for RPB1 and AF-RPB2F (5′-ACGATCCATGGAGTTCCTCA-3′)/AF-RPB2R (5′-CGTTGTACATGACCTCGAAA-3′) for RPB2. Then, 20 μl of PCR mixture consisted of 10 μl of GoTaq master mix (Promega Co., Ltd.), 1 μl of DNA template, 0.5 μl of each primer (10 mM), and 8 μl of sterile distilled water. The PCR conditions included initial denaturation at 95°C for 4 min, 40 cycles of 30 s at 94°C, 30 s at 53°C (annealing temperature), and 50 s at 72°C, with a final elongation at 72°C for 8 min for ITS regions. For the other sequence regions, appropriate annealing temperatures were used as 55°C for EF1/EF2 and AF-RPB2 and 48°C for AF-RPB1. The amplicons were confirmed by running the PCR product on 1% agarose gel with GelRed^TM^ Nucleic Acid Gel Stain (Biotium, Hayward, CA, United States). Furthermore, the PCR products were purified by ExoSAP-IT PCR Product Cleanup reagent (Applied Biosystems^TM^) following the instructions of the manufacturer and sequenced in both directions by using the BigDye Terminator v. 3.1 ready reaction mixture (Perkin-Elmer, Warrington, United Kingdom). Sequence data was obtained on ABI PRISM^TM^ 3100 genetic analyzer (Applied Biosystems, Foster City, CA, United States). They were assembled using CAP3 (Huang and Madan, 1999), combined in species, edited on AliView (Larsson, 2014), and used for phylogenetic analysis.

### Phylogenetic Analyses

Multigene phylogenetic analyses of *Fusarium* spp. were conducted in this study using concatenated DNA sequences of the ITS rDNA, TEF1-α, RPB1, and RPB2 gene regions. The sequences were obtained from the NCBI database for 64 isolates previously used in the AFC phylogenetic analyses (Kasson et al., 2013; Carrillo et al., 2019; Sandoval-Denis et al., 2019; Lynn et al., 2020) (the accession numbers are included in Supplementary Table 2). It was aligned using MUSCLE algorithm (Edgar, 2004) and adjusted manually. The maximum-likelihood tree with the ultrafast bootstrap of 1,000 replicates (Hoang et al., 2018) was inferred using the IQ-TREE program (Nguyen et al., 2015) with the Model Finder option (Kalyaanamoorthy et al., 2017). *Fusarium pseudensiforme* (NRRL 46517) was used as the outgroup.

### Data Analyses

The relative dominance (RD, %) and frequency of occurrence (FO, %) of fungal species isolated from the head, thorax, and abdomen of female adults of *E. interjectus* were calculated using the equations listed below:


$$\text{RD}\left(\%\right)=\frac{\mathrm{Number}\mathrm{of}\mathrm{fungal}\mathrm{isolates}\mathrm{of}\mathrm{each}\mathrm{species}}{\mathrm{Total}\mathrm{number}\mathrm{of}\mathrm{fungal}\mathrm{isolates}\mathrm{of}\mathrm{all}\mathrm{the}\mathrm{species}}\times 100$$

$$\text{FO}\left(\%\right)=\frac{\begin{array}{c} \mathrm{Number}\mathrm{of}\mathrm{beetles}\mathrm{from}\mathrm{which}\mathrm{each}\mathrm{fungal}\mathrm{species}\\ \mathrm{was}\mathrm{isolated} \end{array}}{\mathrm{Total}\mathrm{number}\mathrm{of}\mathrm{the}\mathrm{beetles}\mathrm{used}\mathrm{for}\mathrm{isolation}}\times 100$$

## Results

### Beetle Specimens

In total, 453 beetle specimens (♀: 439; ♂: 14) were collected from fig branches during March 2, 2018–August 20, 2018. The dispersal peak of females and males was observed in April and March, respectively. Based on morphological characterization, all the beetles were identified as *E. interjectus* (Figure 1). In this study, the sex ratio of adults emerging from the galleries was about 31:1 (♀: ♂). This value was much higher than that of Xyleborini ambrosia beetle which has an average ratio of 13:1 (Kirkendall, 1993; Cooperband et al., 2016; Castro et al., 2019; Cognato et al., 2020). A total of 51 adult specimens (female and alive) were randomly selected and used for fungal isolation during March 2, 2018–April 6, 2018 (Supplementary Table 1).

### Fungal Flora

A total of 96 isolates were selected and purified. Of these, 34 isolates were from the head, 31 were from the thorax, and 31 were from the abdomen (Table 2). After morphological categorization, 25 selected isolates were sequenced. Finally, 13 filamentous fungi (*Fusarium kuroshium*, *Arthrinium arundinis*, *Cladosporium cladosporioides*, *Acremonium* sp., *Fusarium decemcellulare*, *Xylariales* sp., *Pithomyces chartarum*, *Roussoella* sp., *Phialophora* sp., *Stachybotrys longispora*, *Paecilomyces formosus*, *Sarocladium implicatum*, and *Bionectria pityrodes*) were identified, and two unknown isolates could also be detected (Table 2).

**TABLE 2:** Relative dominance (RD, %) and frequency of occurrence (FO, %) of fungal species isolated from the head, thorax, and abdomen of female adults of *Euwallacea interjectus* in this study.

Fungal species	Number of fungal isolates and RD^a^ in each body part				Number of beetles from which each fungal species was isolated and FO^b^ in each body part		
			
	Head	Thorax	Abdomen		Head	Thorax	Abdomen
*Fusarium kuroshium*	30 (88)	12 (39)	14 (45)		30 (59)	12 (24)	14 (27)
*Arthrinium arundinis*	1 (3)	1 (3)	3 (10)		1 (2)	1 (2)	3 (6)
*Cladosporium cladosporioides*	1 (3)	3 (10)	1 (3)		1 (2)	3 (6)	1 (2)
*Acremonium* sp.	–	6 (19)	4 (13)		–	6 (12)	4 (8)
*Fusarium decemcellulare*	–	1 (3)	–		–	1 (2)	–
*Xylariales* sp.	–	1 (3)	–		–	1 (2)	–
*Roussoella* sp.	–	–	1 (3)		–	–	1 (2)
*Phialophora* sp.	–	1 (3)	–		–	1 (2)	–
*Stachybotrys longispora*	–	–	1 (3)		–	–	1 (2)
*Sarocladium implicatum*	–	1 (3)	–		–	1 (2)	–
*Bionectria pityrodes*	–	1 (3)	–		–	1 (2)	–
*Pithomyces chartarum*	1 (3)	–	–		1 (2)	–	–
*Paecilomyces formosus*	–	1 (3)	–		–	1 (2)	–
Unknown 1	1 (3)	2 (7)	4 (13)		1 (2)	2 (4)	4 (8)
Unknown 2	–	1 (3)	3 (10)		–	1 (2)	3 (6)
Total number of fungal isolates	34	31	31	Number of beetles tested	51	51	51

*${}^{\text{a}}\text{RD}\left(\%\right)=\frac{\mathrm{Number}\mathrm{of}\mathrm{fungal}\mathrm{isolates}\mathrm{of}\mathrm{each}\mathrm{species}}{\mathrm{Total}\mathrm{number}\mathrm{of}\mathrm{fungal}\mathrm{isolates}\mathrm{of}\mathrm{all}\mathrm{the}\mathrm{species}}\times 100.$*

*${}^{\text{b}}\text{FO}\left(\%\right)=\frac{\mathrm{Number}\mathrm{of}\mathrm{beetles}\mathrm{from}\mathrm{which}\mathrm{each}\mathrm{fungal}\mathrm{species}\mathrm{was}\mathrm{isolated}}{\mathrm{Total}\mathrm{number}\mathrm{of}\mathrm{the}\mathrm{beetles}\mathrm{used}\mathrm{for}\mathrm{isolation}}\times 100.$*

### Phylogenetic Analysis

We obtained each sequence data for FSSC in this study (427 bp for ITS, 380 bp for TEF1, 1,400 bp for RPB1, and 658 bp for RPB2). The sequences of ITS were homologous to those of *F. euwallaceae* and *F. kuroshium*. The sequences of EF1-α genes were homologous to those of *F. kuroshium* (KX262220 and KX262216). The RPB1 sequences were similar to those of *F. kuroshium* and *F. floridanum*, but there were 3-bp differences among them. The RPB2 sequences were identical to those of *Fusarium* spp. (AF13 and AF17) and *F. kuroshium*. In the phylogenetic tree constructed by IQTree, *F. kuroshium* was placed in the distinct clade among the ambrosia fusaria but closely related to *Fusarium* spp. (AF13 and AF14). The number of parsimony informative sites were 9, 13, 69, and 40 bp for ITS, EF1-α, RPB1, and RPB2, respectively.

**FIGURE 2:**
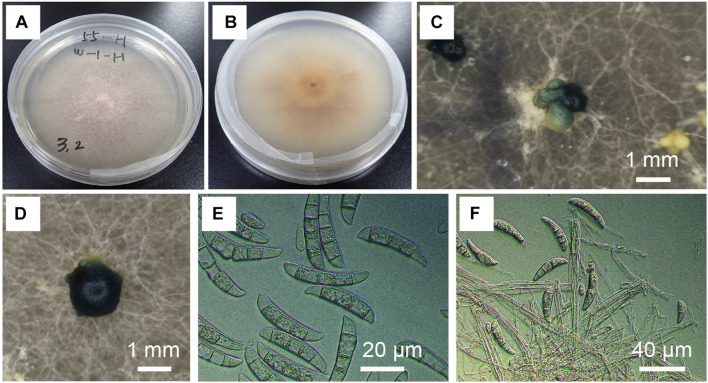
Morphological characteristics of *Fusarium kuroshium*. **(A,B)** Colony on potato dextrose agar (PDA) at 25°C 10 days after inoculation. **(C,D)** Greenish conidial masses formed on sporodochia in culture on PDA (25°C, dark, 2 months). **(E)** Conidial spores in the colony on PDA (25°C, dark, 2 months). **(F)** Conidial spores in the colony on synthetic low-nutrient agar (25°C, dark, 1 month).

**FIGURE 3:**
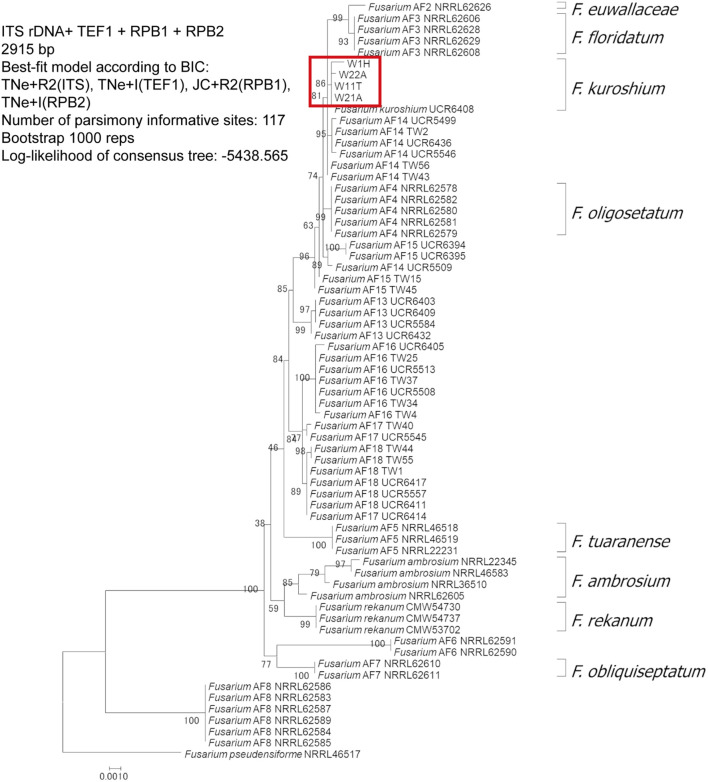
Multilocus phylogenetic analysis of ambrosia *Fusarium* clade conducted with four genes: ribosomal internal transcribed spacer, elongation factor 1-α, DNA-directed RNA polymerase II largest subunit, and DNA-directed RNA polymerase II second largest subunit. The diagram was constructed using IQ-TREE maximum likelihood method bootstrapped with 1,000 replications. The red box indicates the phylogenetic placement of the *Fusarium* species associated with *Euwallacea interjectus* distributed in Hiroshima Prefecture, Western Japan.

*Fusarium kuroshium* was identified based on morphology (Figure 2) and phylogenetic placement (Figure 3). It was characterized by the greenish conidial masses formed on the sporodochia and dolphin-shaped macroconidia, which are typical of ambrosia fusaria associated with *Euwallacea* beetles (Figure 2). It was phylogenetically from the clade of ambrosia *Fusarium* but closely related to *F. kuroshium* (Figure 3).

*Fusarium kuroshium* is problematic in the taxonomy of ambrosia fusaria. It may contain cryptic species or hybrids because their placements were not congruent in the phylogenetic trees constructed by different methods. Species concept of *F. kuroshium* should be re-evaluated using a population genetics approach on ambrosia fusaria.

### Relative Dominance and Frequency of Occurrence

The relative dominance varies from 3 to 88% (Table 2). The vast majority of isolates from the head was *F. kuroshium* with a RD of 88%. The dominance of *F. kuroshium* from the thorax and abdomen was 39 and 45%, respectively (Table 2). The frequency of occurrence of *F. kuroshium* isolated from the head was higher (59%) than that of other fungi and from other body parts (Table 2).

## Discussion

In this study, “symbiotic” relationship is considered as a type of close and long-term biological interaction between ambrosia beetle and its fungal associates. The fungal associates should be stored in its mycangia before release into the galleries and thus significantly dominant among fungal isolates from the mycangia. Our findings showed a novel symbiotic relationship of *E. interjectus*–*F. kuroshium* in fig tree of Japan. To the best of our knowledge, this is the first time to identify this combination in the world. *Fusarium kuroshium* has been found to be closely associated with the oral mycangia (head) of female adults in *E. interjectus* population (Figure 1), suggesting a mycangial fungus possibly with pathogenicity for the fig tree.

This study provided a detailed survey of the different fungal communities associated with the female adults of an ambrosia beetle, *E. interjectus*, among different body parts. We identified fewer fungal species from the head as compared to the thorax and abdomen. Therefore, it could be inferred that the dominance of *F. kuroshium* in the head was higher than that in other parts (Table 2). The previous study showed that the mycangia of *E. interjectus* was located within their oral mycangia as observed by micro-CT scans (Jiang et al., 2019; Figure 1). This fact could provide an explanation for the higher presence of *F. kuroshium* in the head than the thorax and the abdomen because mycangia act as reservoirs to house the fungi during transport (Joseph and Keyhani, 2021).

The *Euwallacea* beetles–*Fusarium* fungi symbiosis has been found in many other countries and regions such as Israel (Mendel et al., 2012), United States (O’Donnell et al., 2015, 2016; Swain et al., 2017), Panama, Costa Rica (Kirkendall and Odegaard, 2007), Taiwan (Carrillo et al., 2019), and has been found to cause a severe disease to some tree species (Aoki et al., 2018, 2019; Table 1). The extensive native range of species in the *Euwallacea* spp. and the results of phylogenetic analyses showed that certain *Euwallacea* species recovered from Florida, United States, and Taiwan were associated with multiple *Fusarium* species from the AFC (O’Donnell et al., 2015; Carrillo et al., 2019). This further suggested that there was a substantial biological variation from native populations of *Euwallacea* spp. and their *Fusarium* spp. associates (Gomez et al., 2018). Some recent studies showed that *Fusarium floridanum* was farmed by *E. interjectus* in box elder (*Acer negundo* L.) in Florida, United States (Aoki et al., 2019). *Fusarium kuroshium* was found to be the most abundant fungal species from macerated heads of *Euwallacea kuroshio* (Kuroshio shot hole borer) which was recovered from the California sycamore (*Platanus racemosa* Nutt.) and avocado (*Persea americana* Mill.) in California, United States (Gomez et al., 2018; Na et al., 2018; Table 1). Our results indicated that *E. interjectus* has an association with an original species of the genus *F. kuroshium* in Japan and may switch to a new fungal symbiont to adapt to newly infested areas. Consistent with a recent study by Carrillo et al. (2019), we hypothesized that a strict relationship exists between the *Euwallacea* spp. and *Fusarium* spp. in the native areas of these organisms. This is in contrast to the promiscuous symbiosis observed in non-native areas (O’Donnell et al., 2015; Stouthamer et al., 2017). Given that several exotic pest insects have switched or gained fungal associates after they were introduced into non-indigenous areas (Carrillo et al., 2014, 2019, 2020), co-phylogenetic analyses were conducted to assess symbiont fidelity within the symbiosis (O’Donnell et al., 2015). Recent studies have also shown that *Euwallacea fornicatus* (Polyphagous Shot Hole Borer) and *E. kuroshio* can survive and reproduce on each other’s symbiotic fungi in their invasive range on artificial media (Carrillo et al., 2020). However, the ability of *E. interjectus* to switch its fungal symbiont is still an unresolved question.

Researchers have devoted over 40 years to resolve the dispersal process of FWD, and only one fungal species, *C. ficicola*, that causes wilt symptom has been discovered in fig orchards in Japan (Kato et al., 1982; Kajitani and Masuya, 2011; Kajii et al., 2013; Morita et al., 2016; Yakushiji et al., 2019). Our initial hypothesis was that *C. ficicola* could be isolated from the wild *E. interjectus* beetle, which emerged from fig trees with FWD. However, the results obtained after fungal isolation indicated another direction since unexpected fungi were collected rather than *C. ficicola*. These results suggested that *C. ficicola* is not infested via the oral mycangia of *E. interjectus*. Under certain conditions, the surface structure of elytra might accidentally trap fungal spores, which are transported by chance to fig trees. *E. interjectus* has numerous pits and setae on its exoskeleton, both of which appear suitable for transporting spores of fungus (Jiang et al., 2019). Our study also displayed that *F. kuroshium*, *A. arundinis*, and *C. cladosporioides* were common in all the three body parts (Table 2). It has been reported in few studies that *F. kuroshium* caused *Fusarium* dieback on woody host species in California, United States (Na et al., 2018). It has been found that *A. arundinis* was a fungal pathogen strain of Korean ginseng root rot in Korea (Durairaj et al., 2018), and *C. cladosporioides* was the causal agent of *Cladosporium* rot in grapes in Chile (Briceño and Latorre, 2008; Mengal et al., 2020). These findings indicated that the unexpected fungi, particularly *F. kuroshium*, possibly take part in FWD in Japan. Inoculation experiments of unexpected fungi on fig trees or saplings have to be conducted to answer this question.

Ambrosia beetles depend upon their fungal symbionts for development and reproduction (Saucedo et al., 2018). Here the *E. interjectus*–*F. kuroshium* symbiotic association seems to exhibit strict specificity based on the dominance of fungal isolates. Thus, *F. kuroshium*, as a potential food source of *E. interjectus*, can play a nutritional role (primary or auxiliary) in this symbiotic system. If so, *E. interjectus* probably avoids the colonization of a branch of stem sections of the fig which are already occupied by the pathogen. Further extensive studies are needed to obtain the evidence which could support the actual function and specificity of this novel symbiotic association.

## Data Availability Statement

The datasets presented in this study can be found in online repositories. The names of the repository/repositories and accession number(s) can be found in the article/Supplementary Material.

## Author Contributions

HK and Z-RJ conceived the study. Z-RJ collected the beetle samples, isolated all examined fungi, analyzed the data, and wrote an early version of the manuscript. HM sequenced all the examined fungi and constructed the phylogenetic tree. HK supervised the study and reviewed the manuscript. All authors contributed to the study and approved the submitted version.

## Conflict of Interest

The authors declare that the research was conducted in the absence of any commercial or financial relationships that could be construed as a potential conflict of interest.

## Publisher’s Note

All claims expressed in this article are solely those of the authors and do not necessarily represent those of their affiliated organizations, or those of the publisher, the editors and the reviewers. Any product that may be evaluated in this article, or claim that may be made by its manufacturer, is not guaranteed or endorsed by the publisher.
