# Supplementary material for: Novel Symbiotic Association Between Euwallacea Ambrosia Beetle and Fusarium Fungus on Fig Trees in Japan
Source: Front Microbiol. 2021 Sep 28;12:725210. doi: 10.3389/fmicb.2021.725210 (PMC8506114; doi:10.3389/fmicb.2021.725210)
Supplement: Supplementary file 1 [file Table_1.DOCX]

Table S1. Background information regarding female adults of *E. interjectus* specimens, which were emerged from branch A or B, used for the fungal isolation

| Date in 2018 | Number of specimens | | Total |
| --- | --- | --- | --- |
|  | Branch A | Branch B |  |
| March 2 | 0 | 4 | 4 |
| March 6 | 4 | 0 | 4 |
| March 9 | 4 | 3 | 7 |
| March 10 | 3 | 1 | 4 |
| March 12 | 2 | 2 | 4 |
| March 13 | 5 | 5 | 10 |
| March 14 | 4 | 8 | 12 |
| April 4 | 1 | 0 | 1 |
| April 6 | 4 | 1 | 5 |
|  |  |  |  |
| Total | 27 | 24 | 51 |
