# Supplementary material for: Novel Symbiotic Association Between Euwallacea Ambrosia Beetle and Fusarium Fungus on Fig Trees in Japan
Source: Front Microbiol. 2021 Sep 28;12:725210. doi: 10.3389/fmicb.2021.725210 (PMC8506114; doi:10.3389/fmicb.2021.725210)
Supplement: Supplementary file 2 [file Table_2.docx]

Table S2. Isolates and sequence data used in this study.

| Isolate | Fungal species | ITS | TEF1 | RPB1 | RPB2 |
| --- | --- | --- | --- | --- | --- |
| NRRL 22345 | *Fusarium ambrosium* (AF-1) | KC691557 | KC691529 | KC691586 | KC691647 |
| NRRL 36510 | *Fusarium ambrosium* (AF-1) | KC691558 | KC691530 | KC691588 | KC691648 |
| NRRL 46583 | *Fusarium ambrosium* (AF-1) | KC691556 | KC691528 | KC691585 | KC691646 |
| NRRL 62605 | *Fusarium ambrosium* (AF-1) | KC691559 | KC691531 | KC691589 | KC691649 |
| TW43 | *Fusarium kuroshium* (AF-12) | MK432868 | MK435445 | MK435497 | MK435529 |
| UCR6408 | *Fusarium kuroshium* (AF-12) | MK432885 | MK435462 | MK435514 | MK435546 |
| NRRL 46517 | *Fusarium pseudensiforme* | KC691584 | KC691555 | KC691615 | KC691674 |
| UCR5584 | *Fusarium* sp. AF-13 | MK432880 | MK435457 | MK435509 | MK435541 |
| UCR6394 | *Fusarium* sp. AF-13 | MK432881 | MK435458 | MK435510 | MK435542 |
| UCR6403 | *Fusarium* sp. AF-13 | MK432883 | MK435460 | MK435512 | MK435544 |
| UCR6409 | *Fusarium* sp. AF-13 | MK432886 | MK435463 | MK435515 | MK435547 |
| UCR6432 | *Fusarium* sp. AF-13 | MK432890 | MK435467 | MK435519 | MK435551 |
| TW2 | *Fusarium* sp. AF-14 | MK432862 | MK435439 | MK435491 | MK435523 |
| TW56 | *Fusarium* sp. AF-14 | MK432872 | MK435449 | MK435501 | MK435533 |
| UCR5499 | *Fusarium* sp. AF-14 | MK432873 | MK435450 | MK435502 | MK435534 |
| UCR5509 | *Fusarium* sp. AF-14 | MK432875 | MK435452 | MK435504 | MK435536 |
| UCR5546 | *Fusarium* sp. AF-14 | MK432878 | MK435455 | MK435507 | MK435539 |
| UCR6436 | *Fusarium* sp. AF-14 | MK432891 | MK435468 | MK435520 | MK435552 |
| TW15 | *Fusarium* sp. AF-15 | MK432861 | MK435438 | MK435490 | MK435522 |
| TW45 | *Fusarium* sp. AF-15 | MK432870 | MK435447 | MK435499 | MK435531 |
| UCR6395 | *Fusarium* sp. AF-15 | MK432882 | MK435459 | MK435511 | MK435543 |
| TW25 | *Fusarium* sp. AF-16 | MK432863 | MK435440 | MK435492 | MK435524 |
| TW34 | *Fusarium* sp. AF-16 | MK432864 | MK435441 | MK435493 | MK435525 |
| TW37 | *Fusarium* sp. AF-16 | MK432865 | MK435442 | MK435494 | MK435526 |
| TW4 | *Fusarium* sp. AF-16 | MK432866 | MK435443 | MK435495 | MK435527 |
| UCR5508 | *Fusarium* sp. AF-16 | MK432874 | MK435451 | MK435503 | MK435535 |
| UCR5513 | *Fusarium* sp. AF-16 | MK432876 | MK435453 | MK435505 | MK435537 |
| UCR6405 | *Fusarium* sp. AF-16 | MK432884 | MK435461 | MK435513 | MK435545 |
| TW40 | *Fusarium* sp. AF-17 | MK432867 | MK435444 | MK435496 | MK435528 |
| UCR5545 | *Fusarium* sp. AF-17 | MK432877 | MK435454 | MK435506 | MK435538 |
| UCR6414 | *Fusarium* sp. AF-17 | MK432888 | MK435465 | MK435517 | MK435549 |
| TW1 | *Fusarium* sp. AF-18 | MK432860 | MK435437 | MK435489 | MK435521 |
| TW44 | *Fusarium* sp. AF-18 | MK432869 | MK435446 | MK435498 | MK435530 |
| TW55 | *Fusarium* sp. AF-18 | MK432871 | MK435448 | MK435500 | MK435532 |
| UCR5557 | *Fusarium* sp. AF-18 | MK432879 | MK435456 | MK435508 | MK435540 |
| UCR6411 | *Fusarium* sp. AF-18 | MK432887 | MK435464 | MK435516 | MK435548 |
| UCR6417 | *Fusarium* sp. AF-18 | MK432889 | MK435466 | MK435518 | MK435550 |
| NRRL 62626 | *Fusarium euwallaceae* (AF-2) | KC691560 | KC691532 | KC691590 | KC691650 |
| NRRL 62606 | *Fusarium floridatum* (AF-3) | KC691561 | KC691533 | KC691591 | KC691651 |
| NRRL 62608 | *Fusarium floridatum* (AF-3) | KC691562 | KC691534 | KC691592 | KC691652 |
| NRRL 62628 | *Fusarium floridatum* (AF-3) | KC691563 | KC691535 | KC691593 | KC691653 |
| NRRL 62629 | *Fusarium floridatum* (AF-3) | KC691564 | KC691536 | KC691594 | KC691654 |
| NRRL 62578 | *Fusarium oligoseptatum* (AF-4) | KC691565 | KC691537 | KC691595 | KC691655 |
| NRRL 62579 | *Fusarium oligoseptatum* (AF-4) | KC691566 | KC691538 | KC691596 | KC691656 |
| NRRL 62580 | *Fusarium oligoseptatum* (AF-4) | KC691567 | KC691539 | KC691597 | KC691657 |
| NRRL 62581 | *Fusarium oligoseptatum* (AF-4) | KC691568 | KC691540 | KC691598 | KC691658 |
| NRRL 62582 | *Fusarium oligoseptatum* (AF-4) | KC691569 | KC691541 | KC691599 | KC691659 |
| NRRL 22231 | *Fusarium tuaranense* (AF-5) | KC691570 | KC691542 | KC691600 | KC691660 |
| NRRL 46518 | *Fusarium tuaranense* (AF-5) | KC691571 | KC691543 | KC691601 | KC691661 |
| NRRL 46519 | *Fusarium tuaranense* (AF-5) | KC691572 | KC691544 | KC691602 | KC691662 |
| NRRL 62590 | *Fusarium* sp. AF-6 strain | KC691574 | KC691546 | KC691604 | KC691664 |
| NRRL 62591 | *Fusarium* sp. AF-6 strain | KC691573 | KC691545 | KC691603 | KC691663 |
| NRRL 62610 | *Fusarium obliquiseptatum* (AF-7) | KC691575 | KC691547 | KC691605 | KC691665 |
| NRRL 62611 | *Fusarium obliquiseptatum* (AF-7) | KC691576 | KC691548 | KC691606 | KC691666 |
| NRRL 62583 | *Fusarium* sp. AF-8 strain | KC691581 | KC691553 | KC691611 | KC691671 |
| NRRL 62584 | *Fusarium* sp. AF-8 strain | KC691582 | KC691554 | KC691612 | KC691672 |
| NRRL 62585 | *Fusarium* sp. AF-8 strain | KC691577 | KC691549 | KC691607 | KC691667 |
| NRRL 62586 | *Fusarium* sp. AF-8 strain | KC691578 | KC691550 | KC691608 | KC691668 |
| NRRL 62587 | *Fusarium* sp. AF-8 strain | KC691579 | KC691551 | KC691609 | KC691669 |
| NRRL 62589 | *Fusarium* sp. AF-8 strain | KC691580 | KC691552 | KC691610 | KC691670 |
| NRRL 22643 | *Fusarium* sp. AF-9 | KC691583 | DQ247628 | KC691613 | KC691673 |
| CMW53702 | *Fusarium rekanum* (AF-19) | MN249095 | MN249152 | ---- | MN249138, MN249109 |
| CMW54737 | *Fusarium rekanum* (AF-19) | MN954359 | MT009961 | ---- | MT009933, MT010013 |
| CMW54730 | *Fusarium rekanum* (AF-19) | MN954353 | MT009960 | ---- | MT009927, MT010007 |
| W1H | *Fusarium kuroshium* (AF-12) | LC637418 | LC637422 | LC637426 | LC637430 |
| W11T | *Fusarium kuroshium* (AF-12) | LC637420 | LC637424 | LC637428 | LC637432 |
| W21A | *Fusarium kuroshium* (AF-12) | LC637421 | LC637425 | LC637429 | LC637433 |
| W22A | *Fusarium kuroshium* (AF-12) | LC637419 | LC637423 | LC637427 | LC637431 |
